# *QuickStats:* Age-Adjusted Percentage* of Adults Aged ≥18 Years Who Have Difficulty Seeing Even When Wearing Glasses,^†^ by Poverty Status^§^ — National Health Interview Survey, United States, 2018^¶^

**DOI:** 10.15585/mmwr.mm6918a4

**Published:** 2020-05-08

**Figure:**
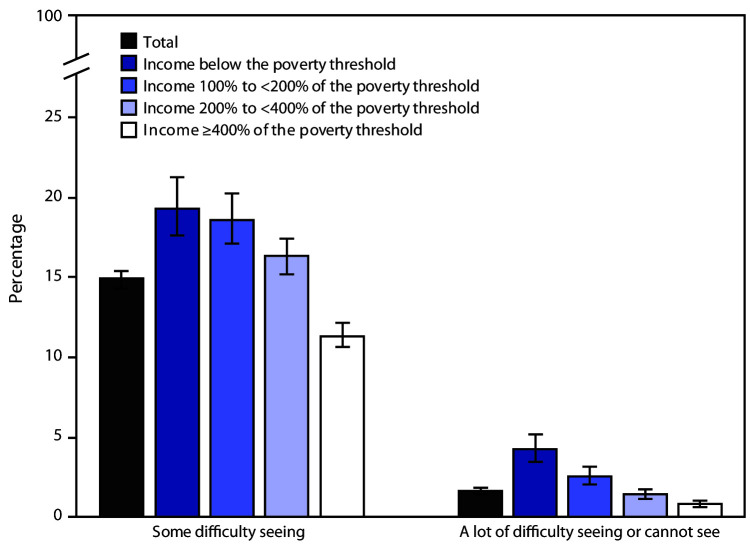
In 2018, 14.9% of adults aged ≥18 years had some difficulty seeing even when wearing glasses, and 1.6% had a lot of difficulty or could not see at all. The percentage of adults who had some difficulty seeing even when wearing glasses decreased as income increased from 19.3% among those with income below the poverty threshold to 11.3% among those with income ≥400% of the poverty threshold. The percentage of adults who had a lot of difficulty or could not see at all also decreased as income increased, from 4.2% among those with income below the poverty threshold to 0.8% among those with income ≥400% of the poverty threshold.

**Source:** National Center for Health Statistics, National Health Interview Survey, 2018. https://www.cdc.gov/nchs/nhis.htm.

